# Low-Dimensional Metal–Organic Magnets as a Route toward the *S* = 2 Haldane Phase

**DOI:** 10.1021/jacs.2c10916

**Published:** 2023-01-10

**Authors:** Jem Pitcairn, Andrea Iliceto, Laura Cañadillas-Delgado, Oscar Fabelo, Cheng Liu, Christian Balz, Andreas Weilhard, Stephen P. Argent, Andrew J. Morris, Matthew J. Cliffe

**Affiliations:** † School of Chemistry, University of Nottingham, University Park, Nottingham, NG7 2RD, United Kingdom; ‡ School of Metallurgy and Materials, University of Birmingham, Elms Road, Edgbaston, Birmingham B15 2TT, United Kingdom; § Institut Laue-Langevin, 71 avenue des Martyrs, CS 20156, 38042 Grenoble, France; ∥ Cavendish Laboratory, Department of Physics, University of Cambridge, JJ Thomson Avenue, Cambridge CB3 0HE, United Kingdom; ⊥ ISIS Neutron and Muon Source, STFC Rutherford Appleton Laboratory, Harwell Oxford, Didcot OX11 0QX, United Kingdom

## Abstract

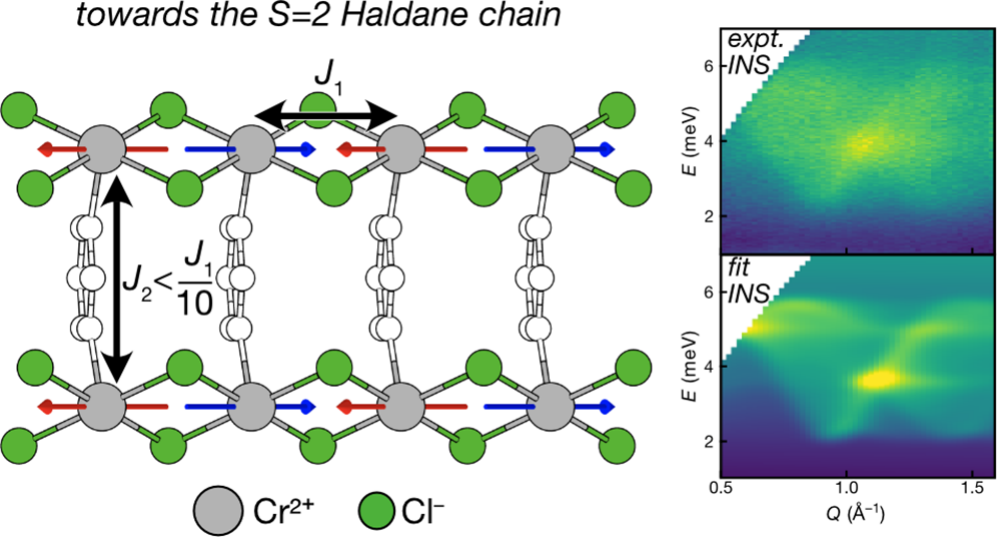

Metal–organic magnets (MOMs), modular magnetic materials where metal atoms are connected by organic linkers, are promising candidates for next-generation quantum technologies. MOMs readily form low-dimensional structures and so are ideal systems to realize physical examples of key quantum models, including the Haldane phase, where a topological excitation gap occurs in integer-spin antiferromagnetic (AFM) chains. Thus, far the Haldane phase has only been identified for *S* = 1, with *S* ≥ 2 still unrealized because the larger spin imposes more stringent requirements on the magnetic interactions. Here, we report the structure and magnetic properties of CrCl_2_(pym) (pym = pyrimidine), a new quasi-1D *S* = 2 AFM MOM. We show, using X-ray and neutron diffraction, bulk property measurements, density-functional theory calculations, and inelastic neutron spectroscopy (INS), that CrCl_2_(pym) consists of AFM CrCl_2_ spin chains (*J*_1_ = −1.13(4) meV) which are weakly ferromagnetically coupled through bridging pym (*J*_2_ = 0.10(2) meV), with easy-axis anisotropy (*D* = −0.15(3) meV). We find that, although small compared to *J*_1_, these additional interactions are sufficient to prevent observation of the Haldane phase in this material. Nevertheless, the proximity to the Haldane phase together with the modularity of MOMs suggests that layered Cr(II) MOMs are a promising family to search for the elusive *S* = 2 Haldane phase.

## Introduction

Metal–organic magnets (MOMs) are assembled from metal nodes bridged by organic molecular linkers into extended networks.^1^ This gives them a number of advantages over conventional inorganic magnets: there is a much wider diversity of organic than atomic ligands;^2^ the modularity of their construction allows for tuning of interactions while retaining the topology;^3^ their longer lengths facilitate magnetic low dimensionality^4,5^ and thus enhanced quantum fluctuations.^6^ Perhaps most excitingly, it has recently been demonstrated that redox-active radical ligands can introduce into MOFs both high electronic conductivity (0.45 S cm^–1^)^7^ and strong magnetic interactions,^8,9^ despite the long distances between metal centers. This suggests that MOMs could form the basis for practical new quantum technology.^10−14^

MOM spin chains are now well-established as host materials for distinctively quantum behavior, from spin fractionalization in Cu(C_6_H_5_COO)_2_·3H_2_O^15^ to the quantum sine-Gordon physics of Cu(pym)(NO_3_)(H_2_O)_2_^16^ (pym = pyrimidine) and [Cu(pym)(H_2_O)_4_]SiF_6_·H_2_O.^17^ One of the most striking quantum discoveries in MOMs was the measurement of the topological Haldane gap in the antiferromagnetic *S* = 1 spin chain MOM Ni(C_2_H_8_N_2_)·2NO_2_(ClO_4_),^18−20^ and subsequent efforts have uncovered a number of other high-quality model systems.^21−24^ The Haldane phase is yet to be experimentally realized for spins *S* > 1.

The difficulty of reaching the Haldane phase for *S* ≥ 2 is largely because the size of the Haldane gap relative to the intrachain exchange, Δ/*J*_1_, decreases significantly from Δ/*J*_1_ = 0.41 for *S* = 1 to Δ/*J*_1_ = 0.087 for *S* = 2, making the gap both more sensitive to the presence of single-ion anisotropy and non-Heisenberg exchange interactions and harder to detect when present.^25^ These challenges have meant that although antiferromagnetic (AFM) *S* = 2 spin chains which could be candidates to host the Haldane phase have been identified, the *S* = 2 gap has not yet been observed.^26−30^ The combination of modularity and the low dimensionality of MOMs means they are an ideal platform to search for the *S* = 2 Haldane phases. However, the most synthetically accessible *S* = 2 transition metal ion is Fe^2+^, which typically possesses large single-ion anisotropy due to its partially quenched ^5^T_2g_ ground state, and other *S* = 2 ions, Mn^3+^ and Cr^2+^, are usually sensitive to reduction or oxidation in ambient conditions. As a result, the chemistry of MOMs which could host *S* = 2 Haldane phases is comparatively underexplored, and their quantum states are thus unrealized.

Here we report CrCl_2_(pym), a new 2D layered magnetic coordination polymer consisting of CrCl_2_ chains bridged by pym ligands. CrCl_2_(pym) has a structure analogous to that of the other transition metal monopyrimidine chlorides (MCl_2_(pym), M = Mn, Fe, Co, Ni, Cu),^31^ the Mn, Co, and Cu analogues of which are reported to possess antiferromagnetic coupling without order down to 1.8 K.^32^ We first describe its synthesis and structural characterization using X-ray diffraction, where the presence of a pronounced Jahn–Teller (JT) distortion confirms the presence of Cr^2+^. We then go on to show using comprehensive magnetic characterization, including bulk magnetization, heat capacity measurements, and powder neutron diffraction (PND) and powder inelastic neutron scattering (INS) measurements of fully protonated samples, that CrCl_2_(pym) orders into a Néel ground state at *T*_N_ = 20.0(3) K, with AFM ordering along the CrCl_2_ chain, FM coupling of the chains through pym, and interlayer FM correlations. Through a detailed analysis of the neutron scattering data, in combination with density-functional-theory (DFT) calculations, we quantitatively determine the size of the key magnetic interactions, which suggest that CrCl_2_(pym) is a well-separated *S* = 2 AFM with near-isotropic single-ion properties. We therefore suggest that through careful ligand choice this family of MOMs offers a potential route to realize the Haldane phase for *S* = 2.

## Results

### Synthesis and Structure

**Figure 1:**
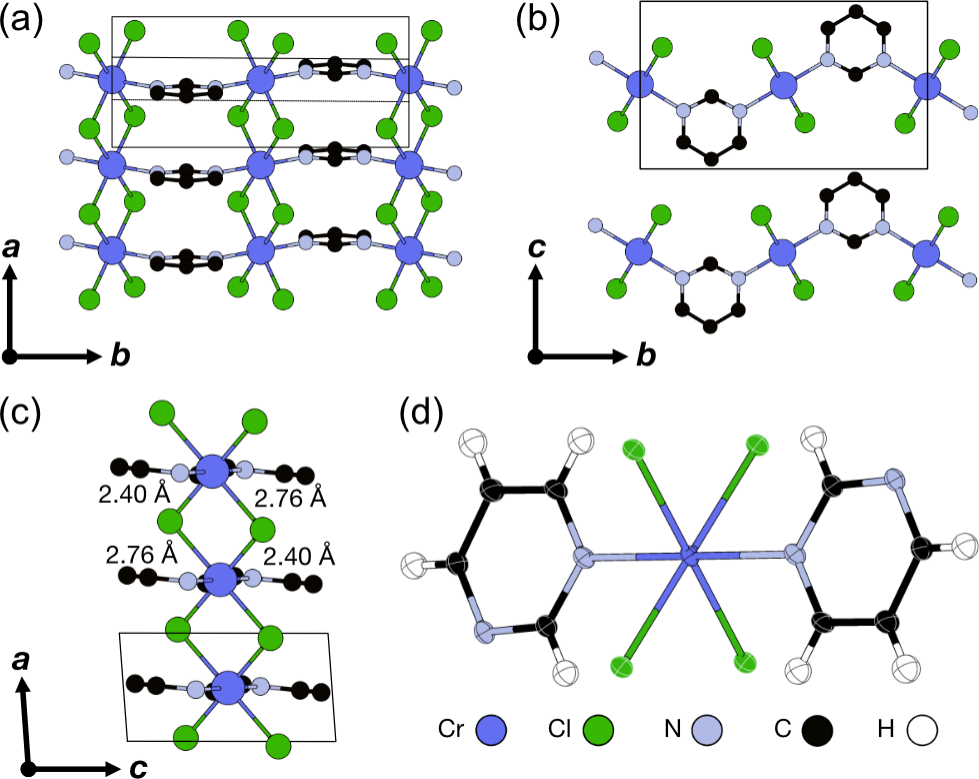
Crystal structure of CrCl_2_(pym) viewed along the (a) *c*, (b) *a*, and (c) *b* axes. Cr–Cl bond lengths are labeled, and H atoms are omitted for clarity. (d) ORTEP diagram showing the coordination environment.

We synthesized CrCl_2_(pym) by reacting CrCl_2_ with pyrimidine. We found that the monopyrimidine CrCl_2_(pym) forms in a wide variety of solvents and stoichiometries, and even via neat combination and with excess ligand, although bispyrimidine analogues are known for other transition metals.^33−35^ Single crystals suitable for X-ray diffraction measurements were grown through vapor diffusion. We solved the structure from single-crystal X-ray diffraction (SCXRD) data and found that CrCl_2_(pym) crystallizes in the monoclinic space group *P*2_1_/*m* with two formula units in the unit cell (Table S1). The Cr^2+^ ions are coordinated by four Cl^–^ ligands and two N atoms from the pyrimidine ligands, which form a distorted CrCl_4_N_2_ octahedron (Figure 1c,d). The chromium octahedra edge share through the Cl^–^ ligands along the crystallographic *a* direction, and these chains are connected by pyrimidine ligands along the crystallographic *b* direction with an alternating orientation to form corrugated layers (Figure 1a). These layers stack in the crystallographic *c* direction through van der Waals interactions (Figure 1b). The Cr^2+^ ion has a large JT distortion, with a long Cr–Cl bond length of *d*_Cr–Cl_ = 2.761(5) Å, comparable to the complex Cr^2+^Cl_2_(pyridine)_4_*d*_Cr–Cl_ = 2.803(1) Å,^36^ confirming the Cr^2+^ oxidation state. Powder X-ray diffraction performed after exposure to air for 1 month show the lattice distortion resulting from this JT distortion is retained, demonstrating that the bulk of the sample maintains the Cr^2+^ oxidation state after exposure to air (Figure S4).

### Magnetic Susceptibility

**Figure 2:**
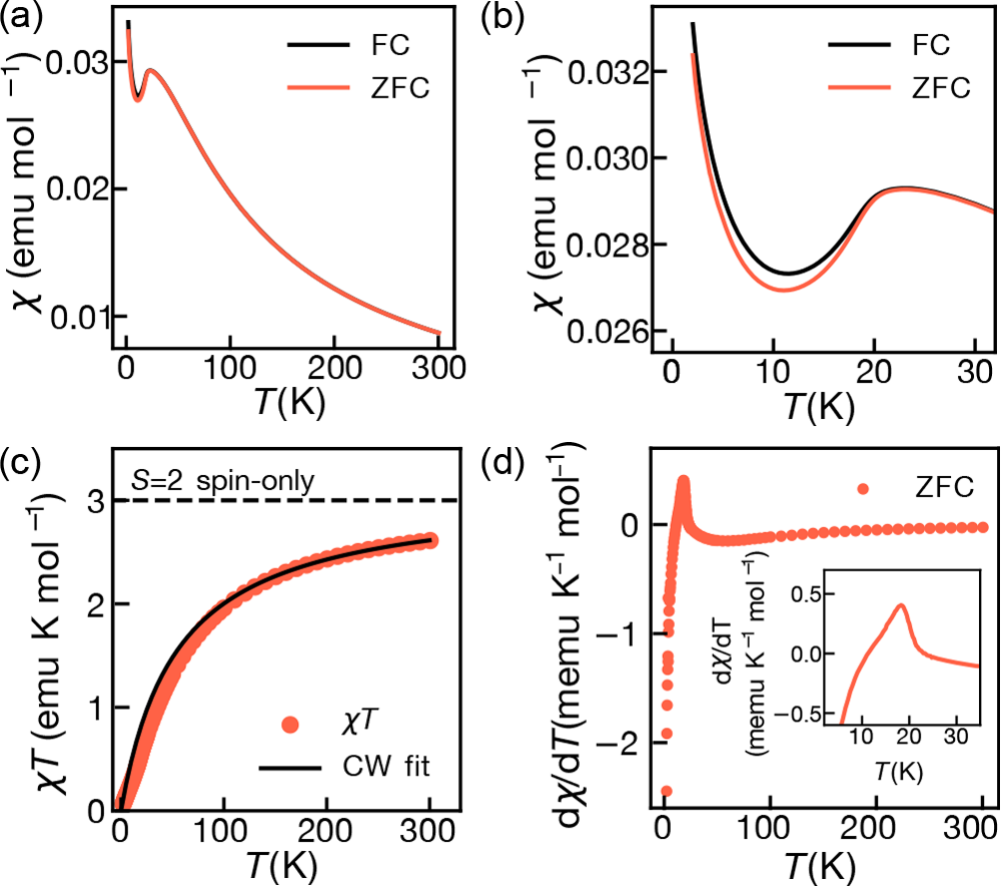
Magnetic susceptibility, χ, measurements of CrCl_2_(pym). (a) χ(*T*) measured in zero field cooled (ZFC) and field cooled (FC) conditions from 2 to 300 K. (b) χ(*T*) data highlighted for 2–30 K. (c) *χT*(*T*) in ZFC and FC conditions for 2–300 K, with Curie–Weiss fit carried out over 300 > *T* > 150 K. Dashed line shows the *S* = 2 spin-only limit. (d) ZFC dχ/d*T*(*T*) over 2–300 K. Inset: ZFC dχ/d*T*(*T*) over 2–35 K.

As we expected CrCl_2_(pym) to be an *S* = 2 2D magnet, we measured its temperature dependent magnetic susceptibility, χ(*T*). The sample was measured under field cooled (FC) and zero field cooled (ZFC) conditions in a 0.01 T dc field from 2 to 300 K. These data show a broad peak at 20–25 K characteristic of short-range ordering and low-dimensional magnetism (Figure 2a). The dχ/d*T*(*T*) data show a discontinuity at 20 K, indicating a phase transition from a disordered magnetic state to a long-range-ordered AFM state (Figure 2d). Fitting χ^–1^(*T*) data to the Curie–Weiss law gave a Curie constant, *C* = 3.08(1) emu K mol^–1^, in good agreement with the presence of high-spin Cr^2+^ (*C* = 3 emu K mol^–1^) (Figure 2c,d). The Curie–Weiss temperature is significant and negative, θ = −54.1(5) K, indicating net antiferromagnetic interactions (Figure 2d), and isothermal magnetization measurements carried out at 2 K show that saturation is not reached at fields of 5 T (Figure S7). While *M*(*H*) is linear in μ_0_*H* > 1 T, there is a small sigmoid feature at μ_0_*H* < 1 T consistent with minor paramagnetic impurities.

The rise in χ(*T*) below *T* = 10 K indicates the presence of small quantities of paramagnetic spins, which we determined to be 1.1(1) spin % from fitting of the Curie-like tail (Figure S13).^37^ This Curie-like tail may be caused by free spins at chain ends or Cr^3+^ formed due to surface oxidation (Figure 2b). Indeed, measurement of the magnetic susceptibility of CrCl_2_(pym) after air exposure showed a large increase in the paramagnetic contribution, 15.0(2) spin % (Figure S6), and X-ray photoelectron spectroscopy (XPS) of this air-exposed sample primarily detected oxidized Cr (Figure S8), with Cr^3+^, Cr^6+^, and metallic Cr present, as well as O 1s peaks consistent with the formation of Cr(OH)_3_.^38^

### Heat Capacity

**Figure 3:**
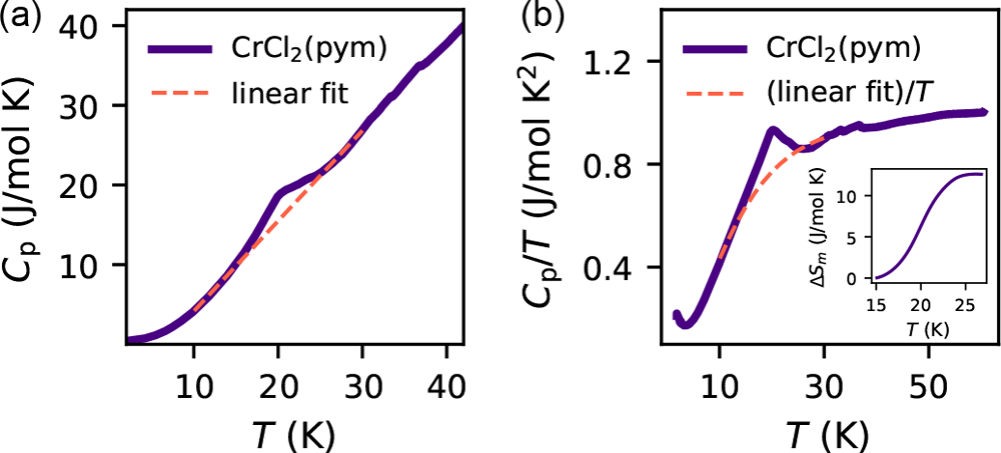
(a) Heat capacity as a function of temperature, *C*_*p*_(*T*), with the nonmagnetic background approximated by a linear fit over the region 10–30 K (dashed line). (b) *C*_*p*_/*T*(*T*), with nonmagnetic background (dashed line). Inset: entropy near *T*_N_.

The molar heat capacity, *C*_*p*_(*T*), of CrCl_2_(pym) was measured from 2 to 60 K. We found a peak in *C*_*p*_(*T*) occurred at 20.0(3) K (Figure 3a), consistent with the magnetic phase transition observed in the magnetic susceptibility data (Figure 2a). We obtained an estimate of the entropy of magnetic ordering by integrating *C*_*p*_/*T*(*T*) after subtraction of a linear background (10–15 and 27–30 K) (Figure 3b), to account for phononic contributions. We found that the measured value of magnetic entropy (*S*_exp._ = 12.7(4) J mol^–1^ K^–1^) is slightly reduced from the expected value (*S*_calc._ = 13.4 J mol^–1^ K^–1^). The small features present in the data between 30 and 40 K are due to instrumental error.

### Neutron Diffraction

**Figure 4:**
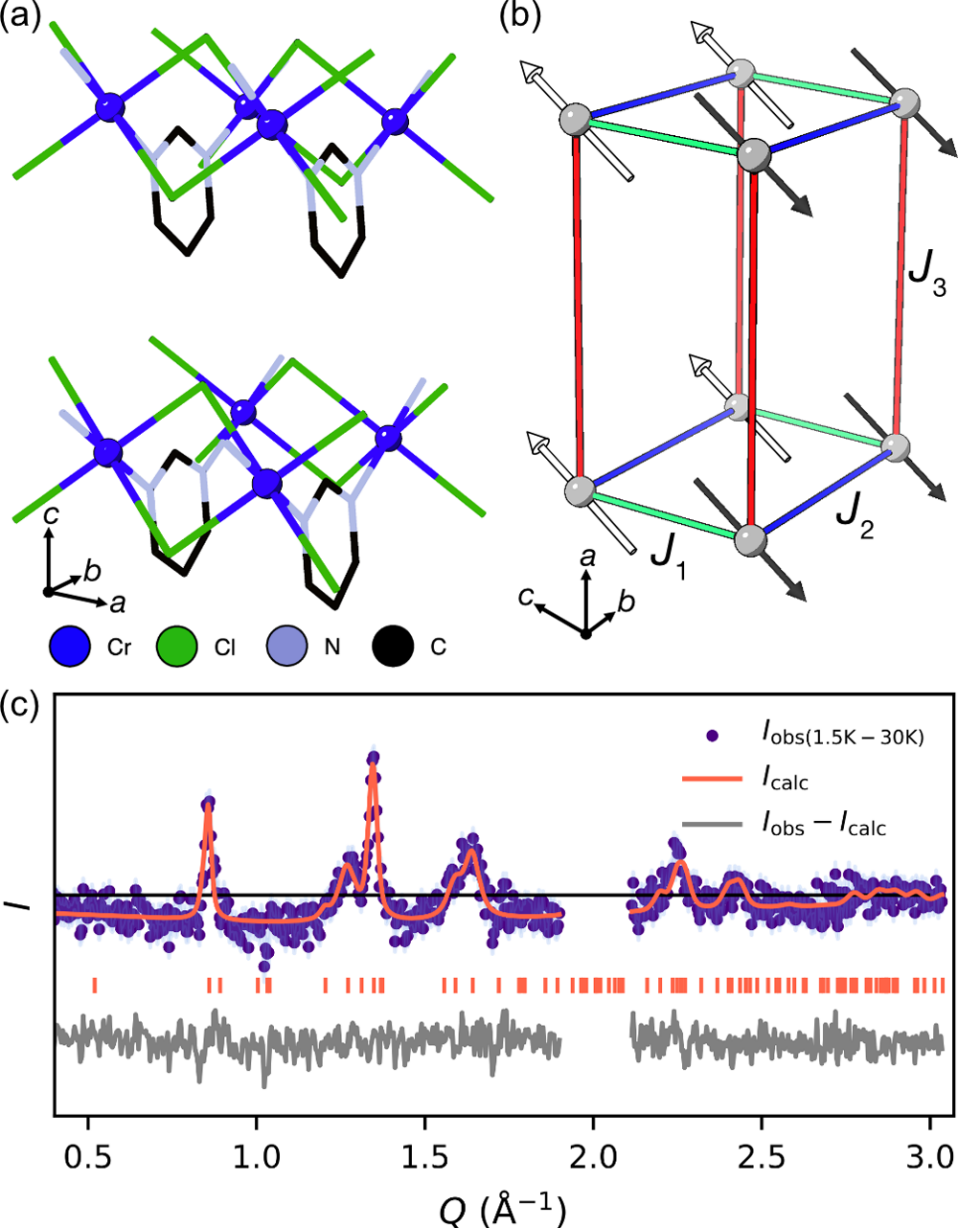
(a) The crystal structure of CrCl_2_(pym), with nuclear axes shown. (b) The magnetic structure, highlighting the three most important exchange interactions, *J*_*n*_, with magnetic axes shown. (c) Rietveld refinement of temperature subtracted neutron scattering data. Data between *Q* = 1.9 and 2.1 Å^–1^ were excluded from the refinement due to incomplete subtraction of nuclear Bragg peaks due to thermal expansion.

Our bulk measurements thus strongly suggested the presence of long-range magnetic order. To determine the nature of this magnetic ground state, we carried out PND using instrument D1B at the Institut Laue-Langevin (ILL) on a 5 g nondeuterated sample of CrCl_2_(pym). We measured the neutron diffraction pattern at two temperatures: *T* = 1.5 K below *T*_N_ and *T* = 30 K above. We isolated the magnetic scattering from instrumental background and nuclear scattering contributions by subtracting the high temperature data set from the low temperature data set (Figure 4c), which allowed us to identify the magnetic Bragg peaks. We were able to index these reflections with a propagation vector **k** = (1/2,0,0), and using symmetry-mode analysis in the ISODISTORT software suite,^39^ we identified there were two possible irreducible representations (irreps), *mY*_1_^–^ and *mY*_2_^–^, in Miller and Love’s notation.^40^ After calibration of the nuclear scale factor through Rietveld refinement of nuclear structure against the high temperature data set, we carried out Rietveld refinement of the magnetic structure using each irrep against the temperature subtracted data set. We found for both nuclear and magnetic refinement that an *hkl*-dependent peak broadening term was necessary to account for the variation in measured peak widths. This showed that only the *mY*_1_^–^ irrep was consistent with experimental data (Figure 4c). The *mY*_1_^–^ irrep lowers the symmetry of the structure to *P*_*c*_2_1_/*c* with the magnetic unit cell relating to the nuclear cell as follows: *a*_mag._ = *c*_nuc._, *b*_mag._ = *b*_nuc._, and *c*_mag._ = 2*a*_nuc._ (Figure 4a,b).

The magnetic structure derived from this refinement is a collinear structure consisting of antiferromagnetically correlated CrCl_2_ spin chains ferromagnetically correlated through the pym ligands, with interlayer ferromagnetic correlations (Figure 4b). The refined magnetic moment for Cr was determined to be *M*_0_ = 2.61(7) μ_B_, significantly less than the spin-only value of *M* = *gS* = 4 μ_B_.

The magnetic moments in our model lie within the *ac* plane; however, components along the *b* direction would be permitted by symmetry. The presence of a component along *b* would result in intensity at the 011_mag._ peak position (*Q* = 1.00 Å^–1^) which is not seen in our data, so any noncollinearity must be small, θ < 8°. The background of this subtracted *I*_1.5 K_ – *I*_30 K_ data set contains a broad negative feature characteristic of magnetic diffuse scattering, which could be modeled by a broad Lorentzian peak centered at the 101_mag._ peak position, with an isotropic correlation length at 30 K of λ = 2.8(2) Å.

### Inelastic Neutron Scattering

**Figure 5:**
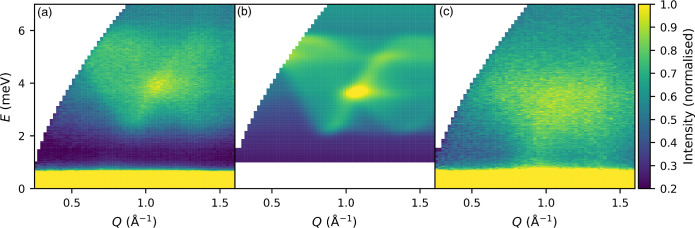
Time-of-flight powder INS spectra of CrCl_2_(pym) with *E*_*i*_ = 12.14 meV measured at (a) 1.7 and (c) 25 K. (b) LSWT calculated scattering intensity fitted to the 1.7 K data, with parameters *J*_1_ = −1.13(4) meV, *J*_2_ = 0.10(2) meV, *J*_3_ = 0.01(1) meV, and *D* = −0.15(3) meV. Hamiltonian described in eq 1.

To measure the parameters of the magnetic Hamiltonian and search for signatures of low-dimensional magnetism, we collected INS spectra on the same powder sample of CrCl_2_(pym) at 1.7 and 25 K using the LET spectrometer at ISIS, using rep-rate multiplication to measure at multiple *E*_*i*_ values simultaneously (*E*_*i*_ = 12.14, 3.70, 1.77 meV). The spectra collected at 1.7 K show a clear excitation centered at Δ*E* = 4.1(2) meV with an energy gap of 2.2(1) meV (Figure 5a) despite the presence of an elevated background due to the incoherent ^1^H scattering. The intensity of this feature rapidly falls with increasing *Q*, until it is masked by phonons, indicating this excitation is magnetic in origin. We were able to quantitatively fit these data using linear spin wave theory (LSWT) (Figure 5b) as implemented by the SpinW software package,^41^ using the following magnetic Hamiltonian:
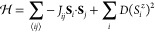
1comprising Heisenberg exchange, *J*_*ij*_, for the three nearest neighbors (i.e., along the CrCl_2_ through the pym ligand and between layers) and a single-ion anisotropy, *D* (Figure 4b). We began by estimating the approximate values for each of *J*_1_, *J*_2_, *J*_3_, and *D* using our bulk magnetic measurements and extrapolating from analogous compounds.^26^ These initial parameters were then optimized using least-squares requirements of the calculated spectrum, including a refined multiplicative scale factor and a background linear in both *Q* and Δ*E*, against the experiment data which gave *J*_1_ = −1.13(4) meV, *J*_2_ = 0.10(2) meV, 0 < *J*_3_ < 0.01(1) meV, and *D* = −0.15(3) meV. The value of *D* was corrected for kinematical consistency,^100^ as by default SpinW uses the inconsistent *D*’ = *D*[1–1/2*S*] = 3/4*D*. A grid search was undertaken to confirm this as a unique solution. Our experimental spectra were consistent with a negligible value for *J*_3_; however, the ground state determined by PND indicates that *J*_3_ must be ferromagnetic. The ratio of *J*_1_/*J*_2_ = 11(2) indicates that the magnetic interactions in this materials are primarily one-dimensional. We therefore decided to investigate the spectrum of CrCl_2_(pym) in the short-range-ordered regime to search for coherent excitations (Figure 5c). Energy cuts, integrated over momentum transfer, 0.76 < *Q* < 1.84 Å^–1^, showed no clear evidence of a gap in the paramagnetic regime, for both *E*_*i*_ = 12.14 meV and *E*_*i*_ = 3.70 meV, suggesting this material is not within the Haldane phase (Figure S3b), although the comparatively high temperature compared to the expected gap size, *T*/Δ = 25, will make this challenging.

### Density-Functional Theory

To understand the origin of the observed low-dimensional interactions, we carried out collinear spin-polarized plane-wave DFT calculations, by exploring the electronic structure of the DFT ground-state spin configuration and calculating the exchange energies using the broken symmetry approach.^42^ We first optimized the geometry of the experimental structure using the PBE functional along with a many-body semiempirical dispersion correction (MBD*)^43^ to describe the weak van der Waals forces between the layers.^44^ We found that this structure was both too dense, with a unit-cell volume of 297.68 Å^3^, 4.8% smaller than the experimental value of 312.75 Å^3^, and lacked the JT distortion characteristic of Cr(II). We therefore included an effective Coulomb on-site energy, *U*_eff_ = *U* – *J*, where *U* is the on-site repulsion and *J* the exchange energy, to account for the overly delocalized Cr d-states. A range of values for *U*_eff_ have been previously explored for Cr, from *U*_eff_ = 2.1 eV to *U*_eff_ = 3.5 eV.^45,46^ We found that *U*_eff_ = 3 eV was able to accurately capture the physics of this system and produced a structure with both a JT distortion and, as a bonus, a volume within +0.2% of experiment.

Exchange interactions were calculated using a 2 × 2 × 1 supercell of the optimized structure (i.e., containing eight distinct Cr atoms) decorated with eight distinct magnetic orderings. Single point energy calculations were then carried out on each configuration, and these DFT+*U* total energies were then fitted to the Hamiltonian described in eq 1 with *D* = 0, i.e., the Heisenberg limit. We carried out these calculations using a series of values of *U*_eff_ to ensure consistency of behavior (Figure S10). For our optimized value of *U*_eff_ = 3 eV, we obtained a self-consistent set of superexchange interactions of *J*_1_ = −2.53(5) meV, *J*_2_ = 0.30(5) meV, and *J*_3_ = −0.09(5) meV. To test the robustness of our DFT+*U* calculations, we performed hybrid calculations using a fraction of Fock exchange as implemented in the HSE functional^47−49^ while maintaining a *U*_eff_ = 3 eV. HSE calculations are computationally expensive due to the calculation of Fock exchange and require the use of norm-conserving pseudopotentials within CASTEP, which limited the sampling of the Brillouin zone and our ability to explore geometry optimizations. Nevertheless, we found that using the HSE functional comparable exchange interactions *J*_1_ = −2.39(1) meV, *J*_2_ = 0.46(1) meV, and *J*_3_ = −0.15(1) meV. These energies are comparable in magnitude to those found experimentally for CrCl_2_(pym) but are notably larger, likely due to the unphysically large degree of delocalization.

**Figure 6:**
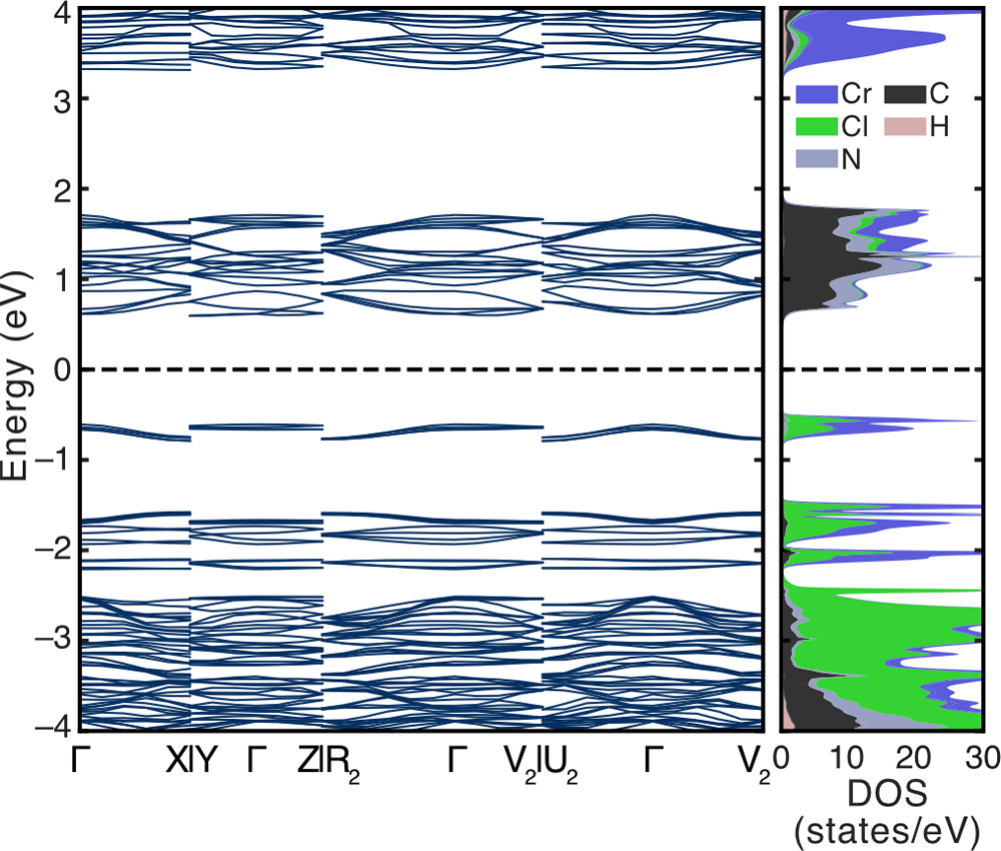
Electronic band structure and projected density of states of the 2 × 2 × 1 supercell using CASTEP and the PBE+*U*+MBD* (*U*_eff_ = 3 eV) functional. The energy zero has been set at the Fermi energy and is shown by the dashed line. The projected density of states has been decomposed by element.

**Figure 7:**
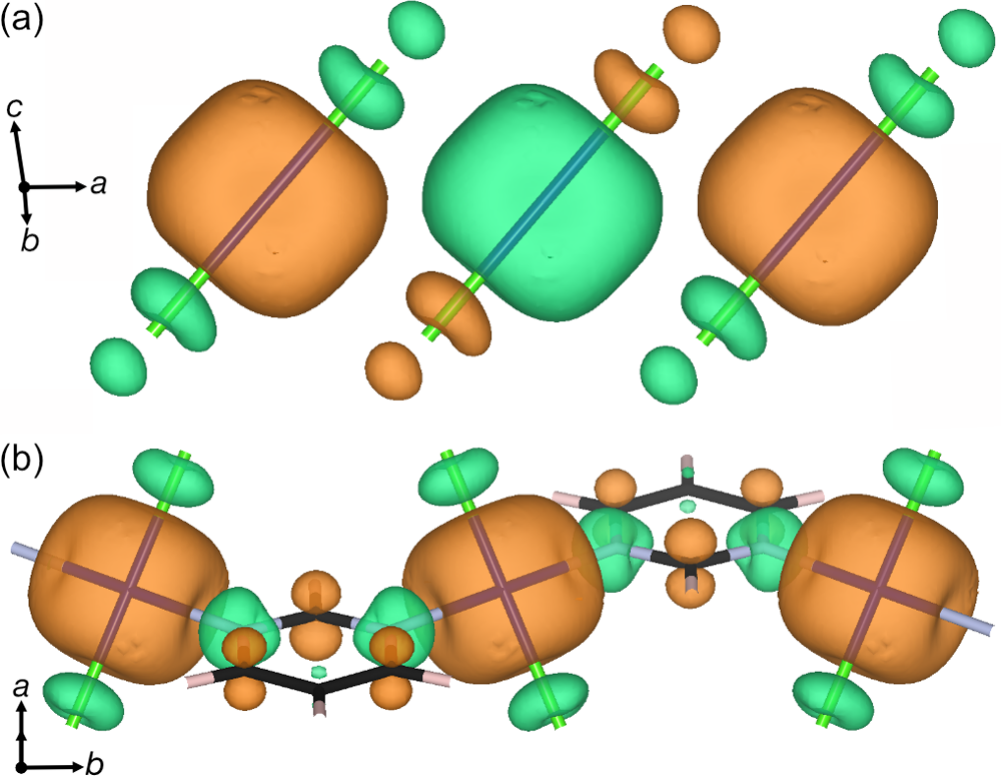
Spin density isosurfaces (0.015 e Å^–3^) highlighting the (a) Cr–Cl chain and (b) Cr–pym chain, derived from our CASTEP PBE+*U*+MBD* (*U*_eff_ = 3 eV) and c2x calculations.^50^

Our calculations allow us not only to predict the interaction energies but also to explore the electronic structure of this material (Figure 6). The predicted thermal band gap is approximately 1.2 eV, and the projection of the DOS onto local orbitals shows that the top of the valence band is broadly Cr and Cl based, while the organic linker pym states are the bottom of the conduction band. This can also be observed in the frontier orbitals, where the HOMO resembles the Cr  orbital antibonding with Cl p orbitals and the LUMO is an antibonding π molecular orbital with a single additional node, suggesting that the lowest lying excitations will be of MLCT character. The spin density is predominantly around the Cr; however, there is significant density on both Cl and pym ligands (Figure 7). Notably, the spin density on pym appears to be primarily of π character and alternates in sign around the ring (Figure 7b).

## Discussion

Metal N-heterocycle dihalides are a diverse family of MOMs, and our study of CrCl_2_(pym) provides one of the most in-depth investigations of the magnetic properties of these materials. There are two common compositions: MX_2_L_2_ and MX_2_L. The monoligand analogues usually contain linear MX_2_ chains and therefore tend to show primarily 1D magnetic behavior, e.g., NiCl_2_(pyrazine) consists of ferromagnetic NiCl_2_ chains antiferromagnetically coupled with *T*_N_ = 10.2 K,^3^ CuCl_2_(pyrazine) is also a very good example of a 1D magnet with no order reported down to 1.8 K, but the strongest interaction in fact occurs through Cu–pyrazine–Cu bridges (*J* = −28 K), due to the JT distortion suppressing exchange in the CuCl_2_ chain.^51^ Preliminary studies of the magnetism of pyrimidine analogues, MCl_2_(pym) (M = Mn, Co, Cu), also detected no magnetic order down to 1.8 K although there are weak AFM interactions present.^32^ The strong interactions, particularly occurring through the CrCl_2_ chain, and magnetic order found in CrCl_2_(pym) are therefore in striking contrast. Additionally, the ferromagnetic exchange we observe occurring through the pym ligand is relatively uncommon for molecular ligands; for example, antiferromagnetic interactions are the norm for pyrazine-bridged MOMs.^52−58^ This ferromagnetic exchange has been previously observed in pym-bridged MOMs, e.g., M(NCS)_2_(pym)_2_ (M = Ni and Co),^59−61^ and has been rationalized by a three-atom π-pathway. Our DFT calculations give further credence to the importance of this pathway.

The bispyrimidine metal chlorides, MCl_2_(pym)_2_ (M = Fe, Co, Ni) and MBr_2_(pym)_2_ (M = Co), unlike most materials in this family, adopt 3D chiral diamondoid structures.^33−35^ MCl_2_(pym)_2_ all magnetically order with canted AFM structures, *T*_N_ = 7.4, 4.7, and 16.3 K for M = Fe, Co, and Ni respectively, likely arising from the interplay between the superexchange interactions and the significant single-ion anisotropy, the principal axes of which are noncollinear.^33^ Bulk susceptibility studies have shown enhancement of *T*_N_ at moderate pressure (Δ*T*_N_/*T*_N_ = 15% at 0.7 GPa),^35^ which suggests that high pressure investigations of Cr-based MOMs may also uncover pressure-switchable magnetic functionality.^62^

The presence of a JT distortion is strong evidence of Cr^2+^, which stands in contrast to the related CrCl_2_(pyrazine)_2_, in which Cr^2+^ spontaneously reduces the ligated pyrazine to a radical anion, thereby dramatically enhancing its conductivity and magnetic superexchange.^8^ The sensitivity of this metal–ligand redox to the coordination sphere is shown by Cr(OSO_2_CH_3_)_2_(pyrazine)_2_, in which Cr remains as Cr^2+^ with a JT distortion.^55^ Studies of molecular complexes have shown this noninnocent behavior is favored by a strong ligand-field environment and a low energy ligand LUMO,^63^ and is consistent with the observed innocence of CrCl_2_(pym), which has both a weaker ligand field than CrCl_2_(pyz)_2_ and a higher energy ligand LUMO (pyrazine, *E*_red_ = +1.10 V and pym *E*_red_ = +0.84 V vs Li/Li^+^).^64^

Our data clearly show that CrCl_2_(pym) has a conventional Néel AFM ground state, *T*_N_ = 20.0(3) K, but also that there is significant magnetic low dimensionality above *T*_N_.

The frustration parameter, , derived from bulk property measurements hints at suppression of magnetic order. As the magnetic lattice does not show an obvious mechanism for geometric frustration, this is likely due to a combination of single-ion anisotropy and low dimensionality arising from the large differences in strength of superexchange in different crystallographic directions.

Additionally, the presence of magnetic diffuse scattering at 30 K not present at 1.5 K indicates the presence of short-range magnetic correlations retained above *T*_N_. Finally, our analysis of the INS spectra shows that the AFM superexchange through the Cr–Cl–Cr bridge is an order of magnitude larger than all other superexchange interactions, .

The importance of low dimensionality can also been seen in the reduction in the apparent size of the Cr^2+^ ordered moment determined via neutron diffraction. The low dimensionality of the structure can reduce the refined moment through disorder, both static short-chain defects and stacking faults^65,66^ and dynamic zero-point fluctuations.^67^ Additionally, as is common in many metal–organic magnets,^68^ there is appreciable delocalization of the spin density onto the ligands, which Mulliken analysis of the DFT-derived electron density suggests is approximately 10%. These factors in combination explain the substantial reduction in the ordered moment (approximately one-third) from that expected moment size, though it is challenging to evaluate their relative contributions.

Despite this low dimensionality, our data indicate that, like other *S* = 2 candidate AFM chains, CrCl_2_(pym) does not show clear Haldane physics. The presence of long-range order at *T*_N_/*J*_1_ = 1.5 hinders observations at low temperatures and the non-negligible single-ion anisotropy (*D* = −0.15(3) meV, *D*/*J*_1_ = 0.13(2)) is sufficient to suppress the Haldane phase, for which the critical value is predicted to be *D*/*J*_1_ = 0.04.^25^ CrCl_2_(pym) is therefore comparable to the other identified candidate *S* = 2 spin chains in both of these parameters,^28−30^ including CrCl_2_,^26^ MnCl_3_(bipy),^27,69^ and CsCrCl_3_,^70^ but none have shown clear evidence of a gapped inelastic neutron spectrum in the disordered phase.

The compound CrCl_2_(pym) is most similar to, both structurally and magnetically, is CrCl_2_,^26^ which also has quasi-1D antiferromagnetic CrCl_2_ chains formed from edge-sharing octahedra (*J*_1_ = −1.13(13) meV, *D* = −0.15(3) meV). However, closer examination reveals significant structural differences that make these magnetic similarities quite surprising. In CrCl_2_(pym) the JT distortion means every superexchange pathway within the CrCl_2_ spin chain passes through a significantly lengthened bond, whereas in CrCl_2_ the equivalent JT distortion lies out of the spin-chain plane and so all Cr–Cl bonds in the chain are short. Superexchange through a JT-lengthened pathway is ordinarily weak, as is indeed found for the direction perpendicular to the CrCl_2_ spin chain in inorganic CrCl_2_, with an order of magntiude weaker exchange *J*_2_ = −0.12(7) meV.

A second distinction between these two compounds is the potential for tuning the interactions through substitution of the ligands. Replacing pyrimidine by a larger bridging ligand may reduce interchain exchange, suppressing long-range order and allowing access to the paramagnetic *S* = 2 quasi-1D AFM at lower temperatures. For example, in NiCl_2_L substituting pyrazine for 1,2-bis(4-pyridyl)ethane reduces *T*_N_ from 10.2 to 5.6 K.^3^ Equally, optimization of the octahedral coordination environment can minimize *D*; for example, in a family of closely related Ni^2+^ compounds, matching of the ligand field strengths reduces the size of the easy-plane anisotropy by a factor of 4.^71^ Our measurements of the INS data already suggest that the interlayer interactions are not significant, but delamination of these van der Waals sheets, as demonstrated for other magnetic metal–organic nanosheets,^72^ may provide an alternative route to better magnetic isolation. These results suggest therefore that bridging CrCl_2_ spin chains with organic ligands may provide promising future candidates for *S* = 2 Haldane chains.

## Conclusion

We have reported the crystal structure, bulk magnetic properties, magnetic ground state, and magnetic excitations of a new coordination polymer, CrCl_2_(pym). We have shown that the oxidation state of chromium in this compound is Cr^2+^, remaining *S* = 2, unlike related CrCl_2_ derived MOMs which undergo redox to form triplet Cr^3+^–radical ligand pairs.^8,63^ CrCl_2_(pym) is found to be a *S* = 2 quasi-one-dimensional antiferromagnet, with an order of magnitude separation in energy scales of superexchange, . However, we did not find clear evidence of the Haldane gap in the disordered phase, suggesting the small *J*_2_ and *D* are sufficient in this compound to either suppress the *S* = 2 Haldane phase or mask it through the stabilization of long-range order. The proximity of CrCl_2_(pym) to the Haldane region of the phase diagram and the modularity inherent to MOMs suggest that optimizing the magnetic properties of these systems, including both superexchange^3^ and single-ion anisotropy,^71^ is a new and promising route to the *S* = 2 Haldane phase.

## Experimental Section

### Synthesis

Synthesis and handling of CrCl_2_(pym) were performed in a dry Ar or N_2_ atmosphere using a MBraun LABstar glovebox or Schlenk line. The reaction of CrCl_2_ (200 mg, 1.63 mmol; Fisher Scientific, 99.9%) and pyrimidine (500 mg, 6.24 mmol; Sigma-Aldrich, ≥98.0%) in 50 mL of methanol (MeOH) rapidly precipitates an orange-brown microcrystalline powder. The CrCl_2_(pym) product was then dried *in vacuo* giving a ca. 90% total yield. The measured (calculated) elemental composition was C, 23.45% (23.67%); H, 1.99% (2.40%); and N, 12.94% (13.80%). This procedure, with quantities scaled up (CrCl_2_, 3.0 g; pyrimidine, 4.0 g; MeOH, 300 mL), was used to synthesize the sample used for neutron-scattering measurements. Crystals of sufficient size for X-ray diffraction studies (127 × 46 × 26 μm) were grown by vapor diffusion of pyrimidine (100 mg, 1.25 mmol) into a concentrated solution of CrCl_2_ in 1 mL of MeOH (10 mg, 0.08 mmol).

### Powder X-ray Diffraction

PXRD data were collected using a PANalytical X’Pert Pro diffractometer equipped with monochromated Cu Kα_1_ radiation (λ = 1.5406 Å). The tube voltage and current were 40 kV and 40 mA, respectively. Scans were performed from 2 to 60° on a zero background silicon crystal plate. Peak fitting and Pawley and Rietveld refinement were performed using Topas Academic v6.^73^

### Single-Crystal X-ray Diffraction

A diffraction-quality single crystal of CrCl_2_(pym) was mounted on a polymer-tipped MiTeGen MicroMountTM using Fomblin (YR-1800 perfluoropolyether oil). The sample was cooled rapidly to 120 K in a stream of cold N_2_ gas, using a Oxford Cryosystems open flow cryostat. Diffraction data were collected on an Oxford Diffraction GV1000 (AtlasS2 CCD area detector, mirror-monochromated Cu Kα radiation source; λ = 1.54184 Å, ω scans). Cell parameters were refined from the observed positions of all strong reflections, and absorption corrections were applied using a Gaussian numerical method with beam profile correction (CrysAlisPro). The structure was solved and refined in Olex2^74^ using SHELXT^75^ and SHELXL,^76^ respectively.

### Magnetic Susceptibility

Magnetic property measurements were carried out on a Quantum Design MPMS superconducting quantum interference device (SQUID). A polycrystalline sample of CrCl_2_(pym) (26.6 mg) was immobilized in eicosane (44.5 mg) and sealed in a low-paramagnetic-impurity borosilicate glass ampule under vacuum. Magnetic susceptibility measurements were performed under field cooled (FC) and zero field cooled (ZFC) conditions in a 0.01 T dc field from 2 to 300 K. Isothermal magnetization measurements were performed at 2 K from 0 T to 5 T to −5 T to 0 T. Data were corrected for the diamagnetism of the sample using Pascal’s constants.^77^

### Heat Capacity

Heat capacity measurements were carried out on a 4.2 mg pellet of CrCl_2_(pym) and silver powder (50 wt %), using a Quantum Design Dynacool physical property measurement system (PPMS), between 2 and 60 K. Apiezon N grease was used to ensure good thermal contact. Contributions to the heat capacity due to Apiezon N were measured separately and subtracted; contributions due to silver were subtracted using tabulated values.^78^

### Powder Neutron Diffraction

PND measurements were carried out on the D1B neutron diffractometer at Institut Laue-Langevin, Grenoble, France. Measurements were collected at 1.5 and 30 K with λ = 2.52 Å between 0.77 and 128.67° with steps of 0.1°. The nuclear structure determined from single-crystal X-ray diffraction was Rietveld refined against neutron diffraction data to evaluate phase purity. Due to the low intensity of magnetic reflections, the magnetic structure was determined by refinement against data from which background and nuclear Bragg peaks were removed by subtraction of data collected at 30 K from those collected at 1.5 K. The magnetic Bragg peaks were indexed to determine the magnetic propagation vector, and then the allowed magnetic irreducible representations were determined using symmetry-mode analysis in the ISODISTORT software.^39^ Using the scale factor determined from Rietveld refinement of the nuclear structure against data at 30 K, and peak parameters determined from Pawley refinement of the nuclear structure against data at 30 K, the direction and magnitude of the ordered moment for the subtracted data set were refined using TOPAS-ACADEMIC 6.0.^73^

### Inelastic Neutron Scattering

Inelastic neutron scattering (INS) measurements were performed on the LET time-of-flight direct geometry spectrometer at ISIS.^79^ The sample (4 g) was contained in a thin aluminum can of diameter 15 mm and height 45 mm and cooled in a helium cryostat. The data were collected at 1.7 and 25 K, for 10 and 7 h, respectively, with *E*_*i*_ = 12.14 meV using the rep-rate multiplication method.^80,81^ The data were reduced using the Mantid-Plot software package.^82^ The raw data were corrected for detector efficiency and time independent background following standard procedures.^83^

### Density-Functional Theory

Plane-wave density-functional-theory calculations were performed using version 19.1 of the CASTEP code.^84^ The Brillouin zone was integrated using a Monkhorst–Pack grid of *k*-points, finer than 2π × 0.05 Å^–1^ spacing.^85^ A Gaussian smearing scheme with a smearing width of 0.20 eV was used during the electronic minimization process. Vanderbilt ultrasoft pseudopotentials were used for computational efficiency (Table S3).^86^ The basis set included plane waves up to an associated kinetic energy of 1100 eV. Geometry optimizations converged until resultant forces were less than 0.05 eV/Å. The OptaDOS postprocessing code was used to integrate individual Kohn–Sham eigenvalues into an electronic density of states,^87^ and the Matador high-throughput environment was used to obtain electronic band structure and density of states plots.^88^

## Data Availability

Additional research data for this article may be accessed at no charge and under CC-BY license at the University of Nottingham Research Data Management Repository 10.17639/nott.7257. Inelastic neutron scattering data measured at ISIS Neutron and Muon Source is available at 10.5286/ISIS.E.RB2090119.

## Supporting Information Available

Information on single-crystal and powder X-ray diffraction, additional inelastic neutron scattering data, powder X-ray and neutron diffraction data, isothermal magnetization measurements, magnetic susceptibility analysis, X-ray photoelectron spectroscopy, transmission electron micrographs, additional details of DFT calculations (PDF)
