# Supplementary material for: Low-Dimensional Metal–Organic Magnets as a Route toward the S = 2 Haldane Phase
Source: J Am Chem Soc. 2023 Jan 10;145(3):1783–92. doi: 10.1021/jacs.2c10916 (PMC9881000; doi:10.1021/jacs.2c10916)
Supplement: Supplementary file 1 — ja2c10916_si_001.pdf [file ja2c10916_si_001.pdf]

Low dimensional metal-organic magnets as a route towards the $S = 2$ Haldane phase

Jem Pitcairn[a], Andrea Iliceto[b], Laura Cañadillas-Delgado[c], Oscar Fabelo[c], Cheng Liu[d], Christian Balz[e], Andreas Weilhard[a], Stephen P. Argent[a], Andrew J. Morris[b], and Matthew J. Cliffe *[a]

[a]School of Chemistry, University of Nottingham, University Park, Nottingham, NG7 2RD, United Kingdom
[b]School of Metallurgy and Materials, University of Birmingham, Birmingham B15 2TT, United Kingdom
[c]Institut Laue-Langevin, 71 avenue des Martyrs - CS 20156, 38042 Grenoble, France
[d]Cavendish Laboratory, Department of Physics, University of Cambridge, JJ Thomson Avenue, Cambridge CB3 0HE, United Kingdom
[e]ISIS Neutron and Muon Source, STFC Rutherford Appleton Laboratory, Harwell Oxford, Didcot OX11 0QX, United Kingdom

December 8, 2022

Contents

List of Figures 2

List of Tables 3

1 Single-crystal X-ray diffraction 4

2 Inelastic neutron spectroscopy 4

*matthew.cliffe@nottingham.ac.uk

List of Figures

S1 Image plot of INS intensity as a function of energy and wave-vector transfer for $CrCl_2$(pym) at: (a) $E_i = 12.14$ meV, $T = 1.7$ K; (b) $E_i = 12.14$ meV, $T = 25$ K; (c) $E_i = 3.70$ meV, $T = 1.7$ K; (d) $E_i = 3.70$ meV, $T = 25$ K; (e) $E_i = 1.77$ meV, $T = 1.7$ K; (f) $E_i = 1.77$ meV, $T = 25$ K. 5

S2 Image plot of INS intensity as a function of energy and wave-vector transfer for $CrCl_2$(pym) at $T = 1.7$ K for (a) $E_i = 3.70$ meV and (b) $E_i = 1.77$ meV. (c) Slice plot of INS intensity at $E_i = 3.70$ meV as a function of energy averaged from $Q = 0.76$ Å^{-1} to $Q = 1.84$ Å^{-1} (left, red). Slice plot of INS intensity at $E_i = 1.77$ meV as a function of wave-vector averaged from $Q = 0.30$ Å^{-1} to $Q = 1.50$ Å^{-1} (right, blue). 6

S3 Image plot of INS intensity as a function of energy and wave-vector transfer for $CrCl_2$(pym) at $T = 25$ K for (a) $E_i = 3.70$ meV and (b) $E_i = 1.77$ meV. (c) Slice plot of INS intensity at $E_i = 3.70$ meV as a function of energy averaged from $Q = 0.76$ Å^{-1} to $Q = 1.84$ Å^{-1} (left, red). Slice plot of INS intensity at $E_i = 1.77$ meV as a function of wave-vector averaged from $Q = 0.30$ Å^{-1} to $Q = 1.50$ Å^{-1} (right, blue). 6

S4 Rietveld refinement[1] of powder X-ray Cu Kα_1 radiation data ($\lambda = 1.5406$ Å) of an air exposed (1 month) sample of $CrCl_2$(pym). The unit cell parameters were allowed to refine freely, however the atomic coordinates and displacement parameters were fixed to those determined from single-crystal XRD. See Table S2 for details. 7

S5 Rietveld refinement[1] of powder neutron diffraction data collected at 30 K and $\lambda = 2.52$ Å of $CrCl_2$(pym). The unit cell parameters and anisotropic displacement parameters were allowed to refine freely, however the atomic coordinates were fixed to those determined from single-crystal XRD. 7

S6 The magnetic susceptibility of $CrCl_2$(pym) under zero-field cooled (ZFC) and field cooled (FC) conditions after 100 days exposed to air from (a) 2 to 300 K and (b) 2 to 30 K. 8

S7 (a) Isothermal magnetisation of $CrCl_2$(pym) measured at 2 K between –5 T to 5 T and (b) its field derivative. 8

S8 X-ray photoelectron spectroscopy data, Shirley background and fitted peaks at binding energies characteristic of the Cr 2p orbitals after exposure to air for 130 days. Spectra were taken from six distinct surface regions on a single powder sample. Spectra (a) and (b) are two of these six spectra gathered, they were chosen to be a representative sample of all six spectra gathered. 9

S9 Transmission electron microscopy (TEM) (a) images of $CrCl_2$(pym) crystallites and (b) EDX spectrum of $CrCl_2$(pym), spotted onto a Cu-based TEM grid. 9

S10 Superexchange interactions J_n along the three shortest Cr-Cr distances with respect to the Hubbard U value. The figure presents both the DFT results calculated with PBE+U and MBD*, as well as the powder INS experimental results fitted using linear spin wave theory. 10

S11 Graphical visualisation of the HOMO and LUMO of the $2 \times 2 \times 1$ supercell calculated at the Gamma point using the DFT package CASTEP along with c2x.[2] The yellow and blue colours correspond to the two different spin channels. 10

S12 PBE+U+MBD* (U=3eV) electronic band structure and projected density of states of the $2 \times 2 \times 1$ supercell. The Fermi energy has been placed at the origin and is shown by the dashed line. The projected density of states has been decompose onto the atomic species and their respective angular momentum channels. 11

S13 The zero-field cooled susceptibility data, $\chi_{\mathrm{exp.}}$, are shown here in blue. The Curie-like tail in these data were fit below $T = 10$ K by an additive Curie and mean-field theory function, $\chi_{\mathrm{C}} + \chi_{\mathrm{MFT}}$, shown in green.[3] The Curie component from this fit was then subtracted over $T = 2$ K to $T = 300$ K, $\chi_{\mathrm{exp.}} - \chi_{\mathrm{C}}$, to show the contribution of short range correlations to $\chi(T)$ more clearly (black). The mean-field theory function component of the fit, χ_{MFT}, is shown in red. 11

List of Tables

S1 Crystal data and single-crystal XRD structural refinement parameters. Bonds lengths for $CrCl_2$(pym). *See Section 1. 4
S2 Refined lattice parameters from powder XRD. 12
S3 Summary of PBE+U+MBD* geometry optimisation results using two different pseudo-potentials and a range of Hubbard U values. Ultrasoft pseudo-potentials have been used as well as NCP19 Vanderbilt (ONCVPSP) "on-the-fly" optimized norm-conserving pseudo-potentials. A cut-off energy of 1100 eV has been used with 18 k-points and a Gaussian smearing scheme with a 0.2 eV smearing width. The Broyden density-mixing scheme has been used throughout the optimisations. 13

1 Single-crystal X-ray diffraction

The diffraction data was processed as a two-component twin using CrysAlisPro 1.171.40.67a (Rigaku OD, 2019) which output hklf4 and hklf5 reflections files. The structure was solved with the hklf4 data and refined as a two component twin against the hklf5 data with the batch scale factor refined to a value of 0.463(3). The R_{int} values for data refined against twinned hklf5 data are artificially large and not meaningful. H atoms were identified in the difference map and refined using riding constraints. H atoms were identified in the difference map and refined using riding constraints.

2 Inelastic neutron spectroscopy

In addition to the spin-wave dispersions (Fig. S1a), we identified a low energy excitation band in our INS spectra at *circa* 0.6 meV (Fig. S1(c), S1(e), S2). These excitations are likely magnetic in origin and related to the long-range ordered phase as they are present below $T_{\mathrm{N}} = 20.0(3)$ K, at $T = 1.7$ K, and absent above, at $T = 25$ K (Fig. S1(d), S1(f), S3). The intensities of the spectra collected at $E_i = 1.77$ meV are lower compared to $E_i = 3.70$ meV due to lower flux at this energy repetition.

Table S1: Crystal data and single-crystal XRD structural refinement parameters. Bonds lengths for $CrCl_2$(pym). *See Section 1.

MW (g mol^{-1})	202.99
Crystal system	Monoclinic
Space group	$P\,1\,2_1/m\,1$
a (Å)	3.6688(2)
b (Å)	12.1030(5)
c (Å)	7.0628(3)
$\beta(°)$	94.236(4)
V (Å^3)	312.76(3)
T (K)	120(17)
Z	2
R_{int}	n/a*
R_1	3.05
wR_2	9.05
GOF	1.034
$\Delta\rho_{\mathrm{max}}$,$\Delta\rho_{\mathrm{min}}$/e Å^{-3}	0.5, -0.4

Bond length	r (Å)
Cr-Cl	2.3952(4)
Cr-Cl	2.761(5)
Cr-N	2.1390(15)
N-C1	1.337(2)
N-C2	1.351(3)
C2 C3	1.378(2)

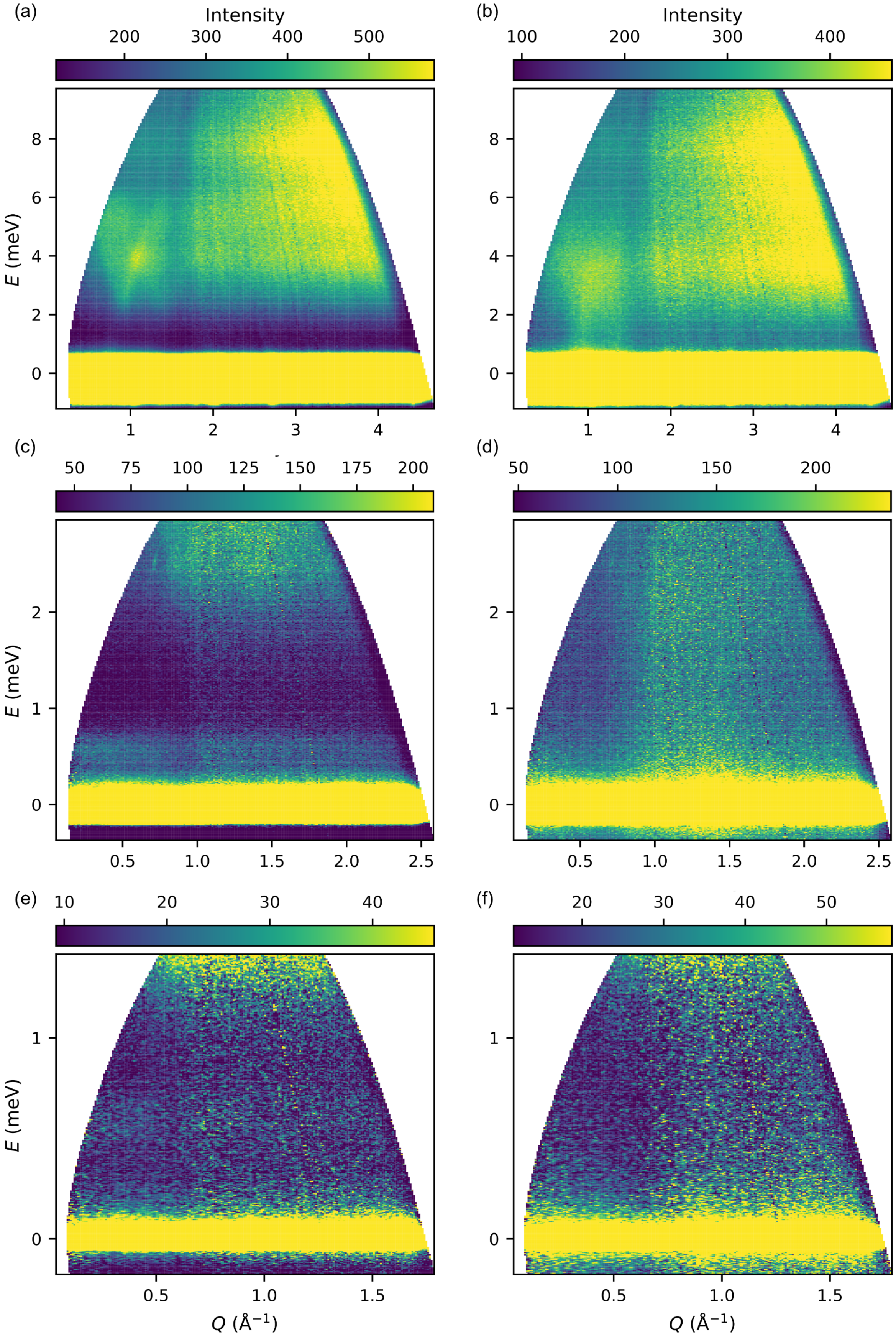

Figure S1: Image plot of INS intensity as a function of energy and wave-vector transfer for $CrCl_2$(pym) at: (a) $E_i = 12.14$ meV, $T = 1.7$ K; (b) $E_i = 12.14$ meV, $T = 25$ K; (c) $E_i = 3.70$ meV, $T = 1.7$ K; (d) $E_i = 3.70$ meV, $T = 25$ K; (e) $E_i = 1.77$ meV, $T = 1.7$ K; (f) $E_i = 1.77$ meV, $T = 25$ K.

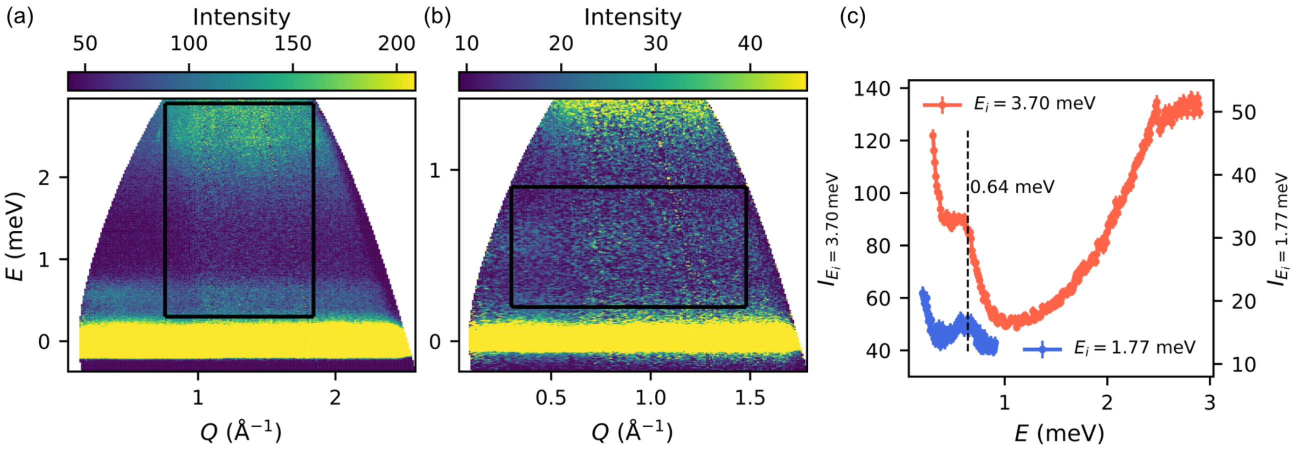

Figure S2: Image plot of INS intensity as a function of energy and wave-vector transfer for $CrCl_2$(pym) at $T = 1.7$ K for (a) $E_i = 3.70$ meV and (b) $E_i = 1.77$ meV. (c) Slice plot of INS intensity at $E_i = 3.70$ meV as a function of energy averaged from $Q = 0.76$ Å^{-1} to $Q = 1.84$ Å^{-1} (left, red). Slice plot of INS intensity at $E_i = 1.77$ meV as a function of wave-vector averaged from $Q = 0.30$ Å^{-1} to $Q = 1.50$ Å^{-1} (right, blue).

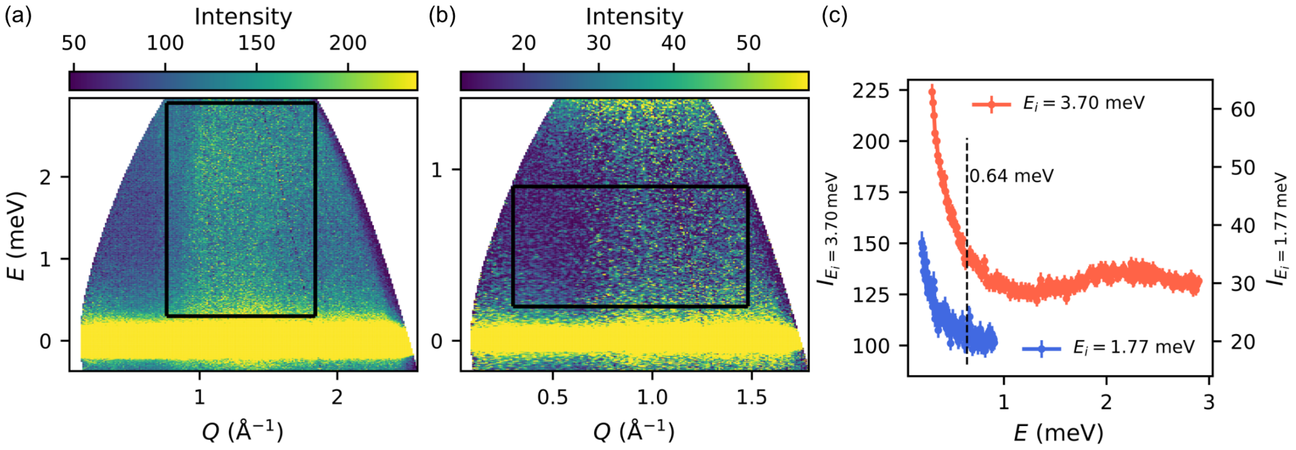

Figure S3: Image plot of INS intensity as a function of energy and wave-vector transfer for $CrCl_2$(pym) at $T = 25$ K for (a) $E_i = 3.70$ meV and (b) $E_i = 1.77$ meV. (c) Slice plot of INS intensity at $E_i = 3.70$ meV as a function of energy averaged from $Q = 0.76$ Å^{-1} to $Q = 1.84$ Å^{-1} (left, red). Slice plot of INS intensity at $E_i = 1.77$ meV as a function of wave-vector averaged from $Q = 0.30$ Å^{-1} to $Q = 1.50$ Å^{-1} (right, blue).

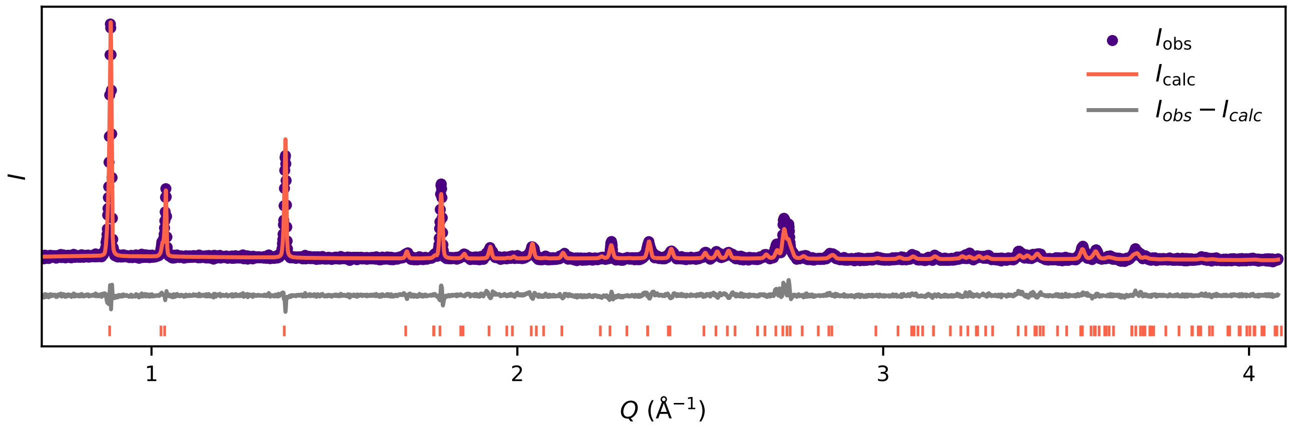

Figure S4: Rietveld refinement[1] of powder X-ray Cu Kα_1 radiation data ($\lambda = 1.5406$ Å) of an air exposed (1 month) sample of $CrCl_2$(pym). The unit cell parameters were allowed to refine freely, however the atomic coordinates and displacement parameters were fixed to those determined from single-crystal XRD. See Table S2 for details.

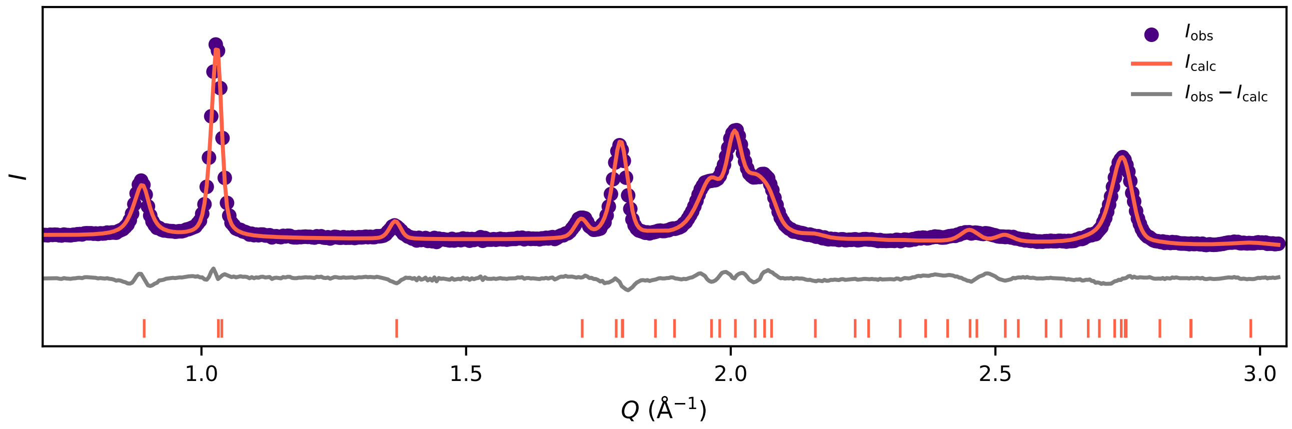

Figure S5: Rietveld refinement[1] of powder neutron diffraction data collected at 30 K and $\lambda = 2.52$ Å of $CrCl_2$(pym). The unit cell parameters and anisotropic displacement parameters were allowed to refine freely, however the atomic coordinates were fixed to those determined from single-crystal XRD.

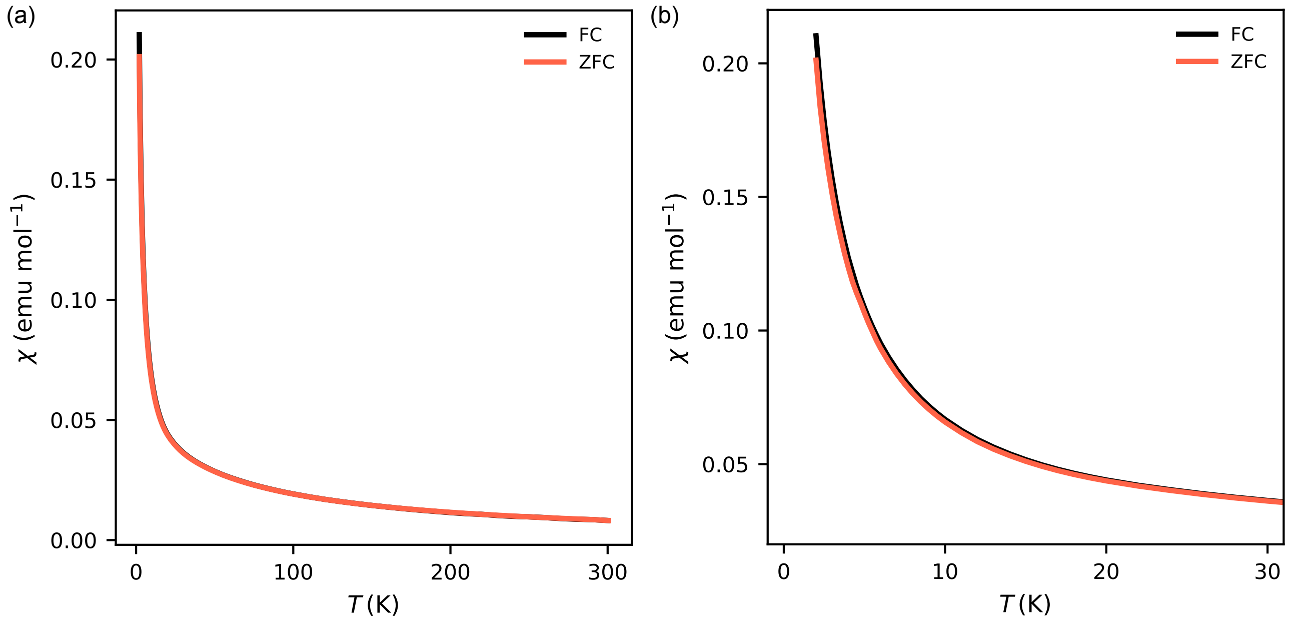

Figure S6: The magnetic susceptibility of $CrCl_2$(pym) under zero-field cooled (ZFC) and field cooled (FC) conditions after 100 days exposed to air from (a) 2 to 300 K and (b) 2 to 30 K.

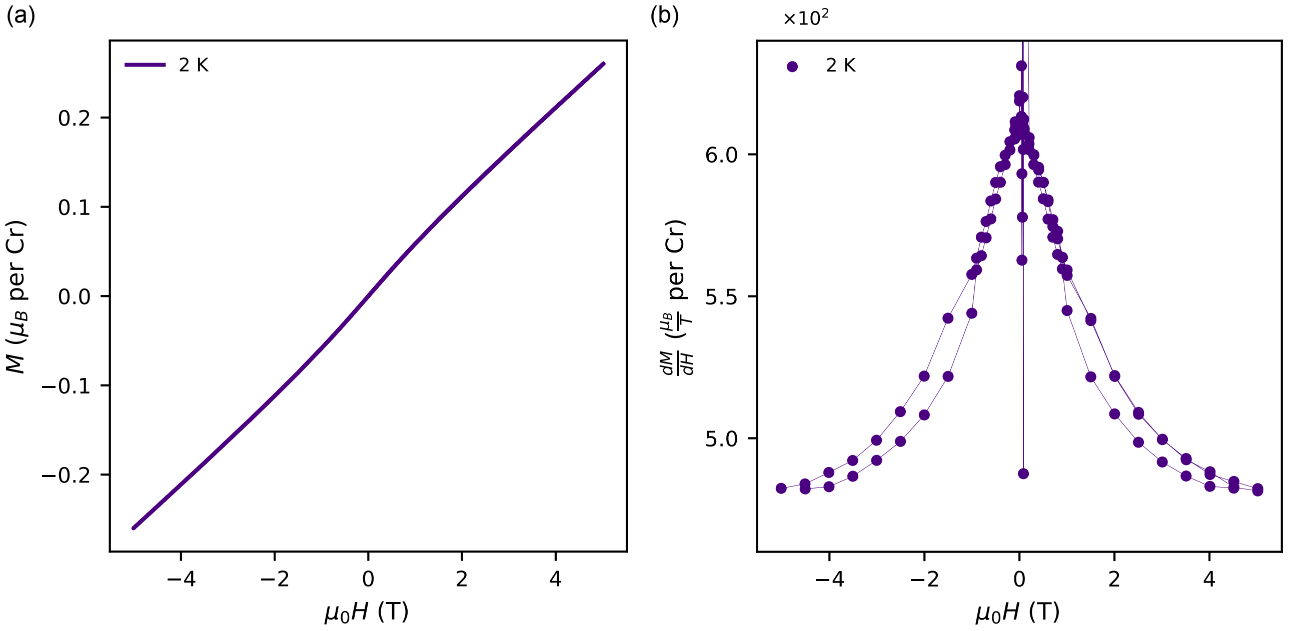

Figure S7: (a) Isothermal magnetisation of $CrCl_2$(pym) measured at 2 K between –5 T to 5 T and (b) its field derivative.

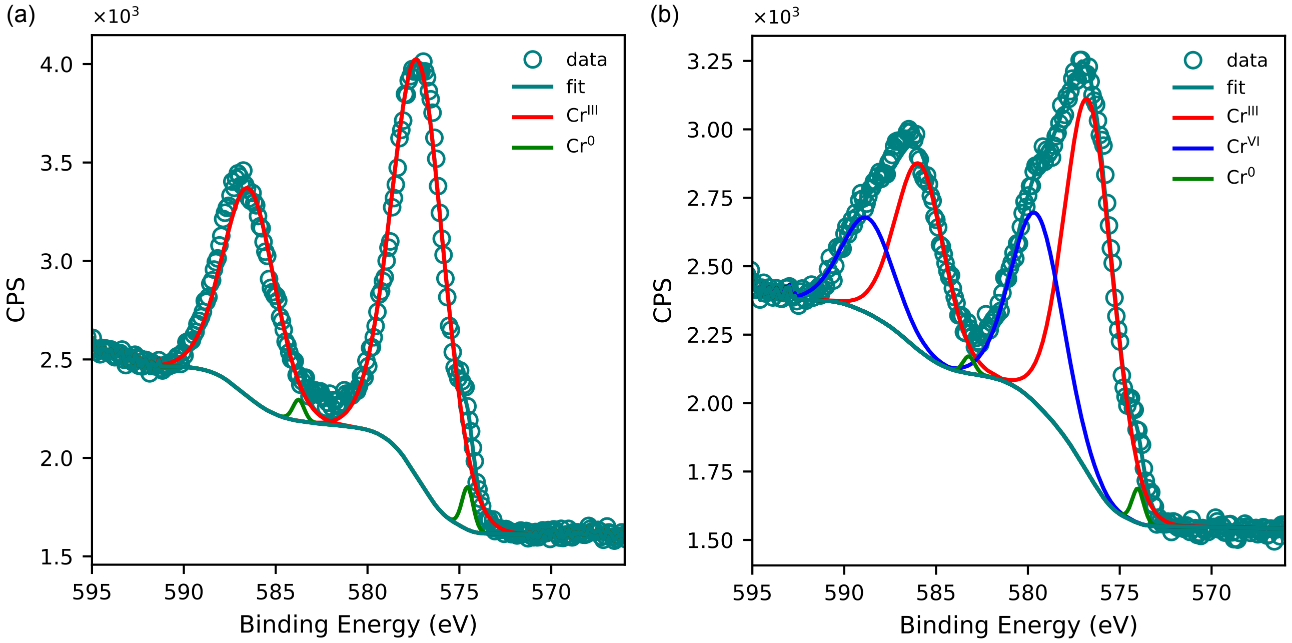

Figure S8: X-ray photoelectron spectroscopy data, Shirley background and fitted peaks at binding energies characteristic of the Cr 2p orbitals after exposure to air for 130 days. Spectra were taken from six distinct surface regions on a single powder sample. Spectra (a) and (b) are two of these six spectra gathered, they were chosen to be a representative sample of all six spectra gathered.

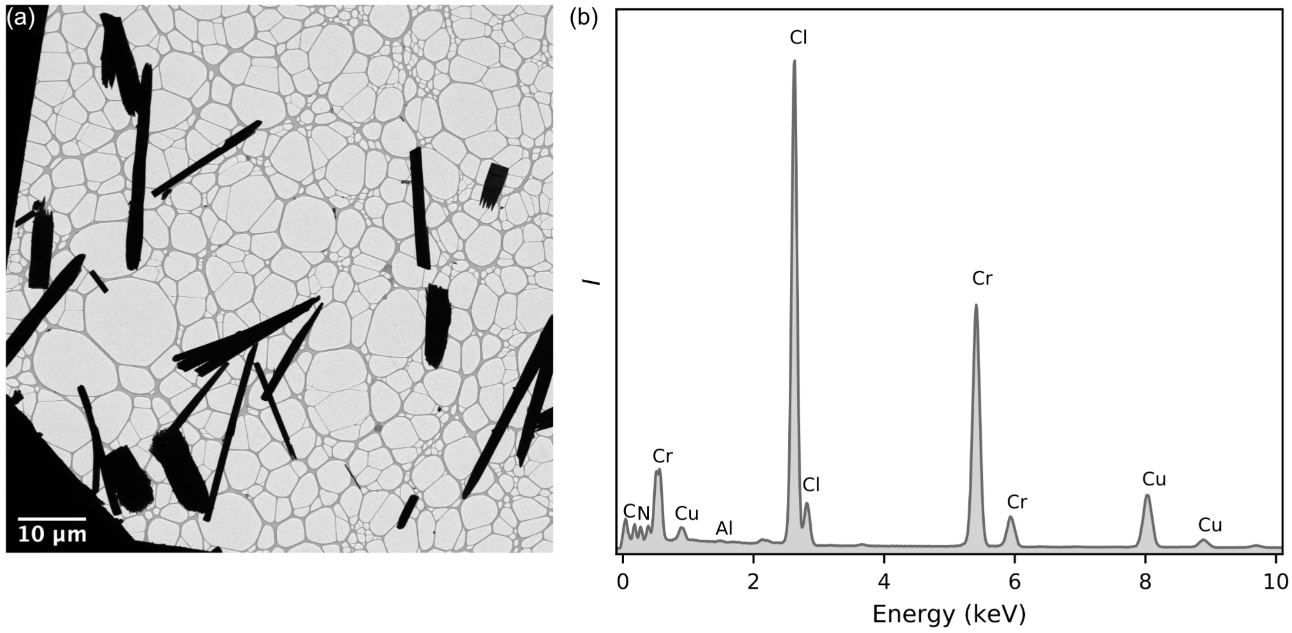

Figure S9: Transmission electron microscopy (TEM) (a) images of $CrCl_2$(pym) crystallites and (b) EDX spectrum of $CrCl_2$(pym), spotted onto a Cu-based TEM grid.

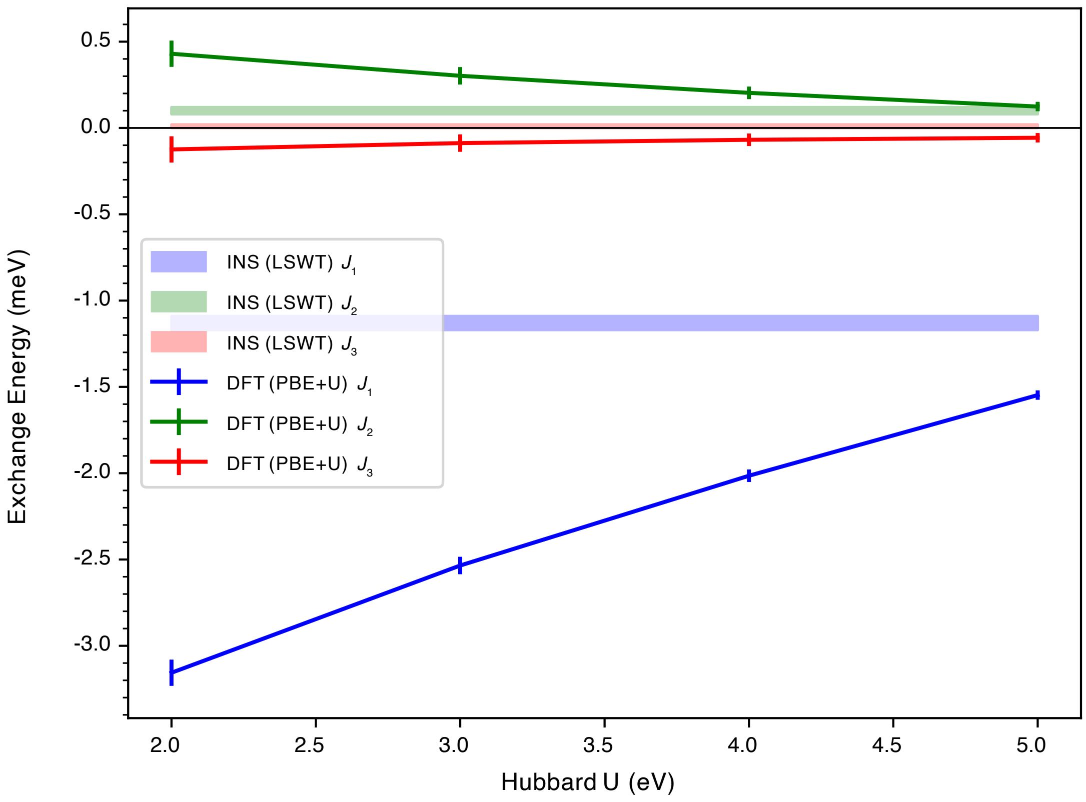

Figure S10: Superexchange interactions J_n along the three shortest Cr-Cr distances with respect to the Hubbard U value. The figure presents both the DFT results calculated with PBE+U and MBD*, as well as the powder INS experimental results fitted using linear spin wave theory.

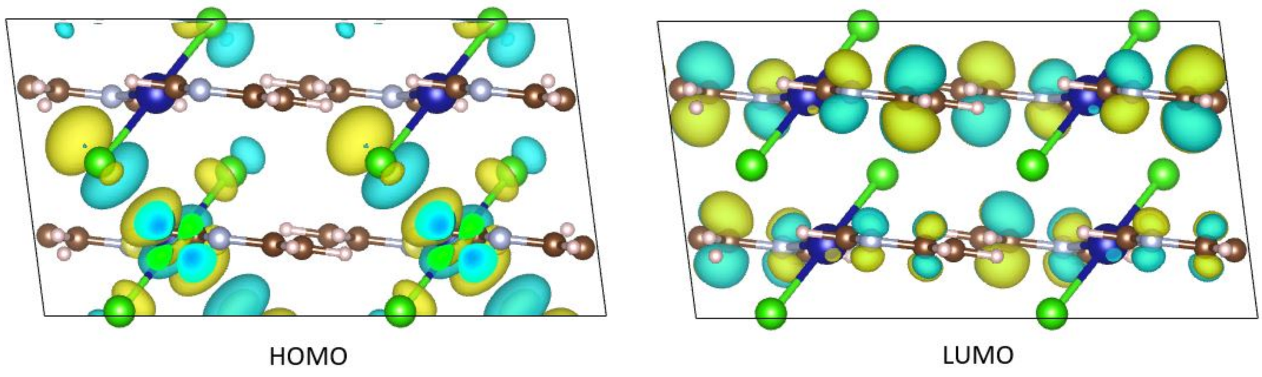

Figure S11: Graphical visualisation of the HOMO and LUMO of the $2 \times 2 \times 1$ supercell calculated at the Gamma point using the DFT package CASTEP along with c2x.[2] The yellow and blue colours correspond to the two different spin channels.

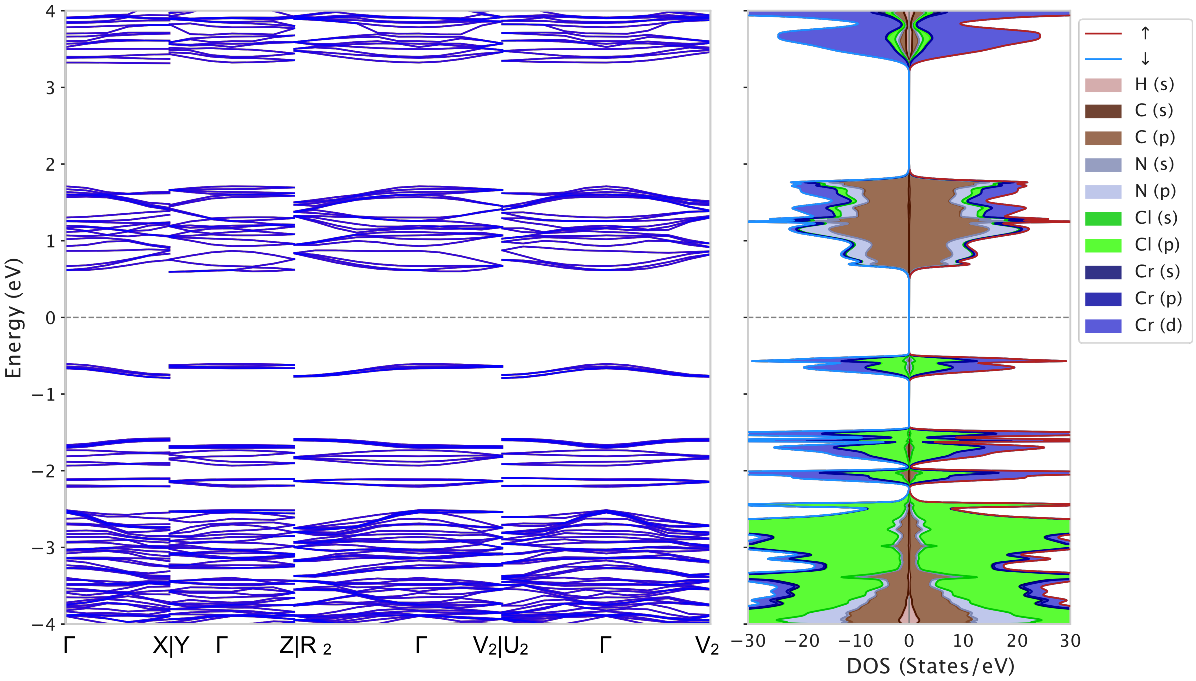

Figure S12: PBE+U+MBD* (U=3eV) electronic band structure and projected density of states of the $2 \times 2 \times 1$ supercell. The Fermi energy has been placed at the origin and is shown by the dashed line. The projected density of states has been decompose onto the atomic species and their respective angular momentum channels.

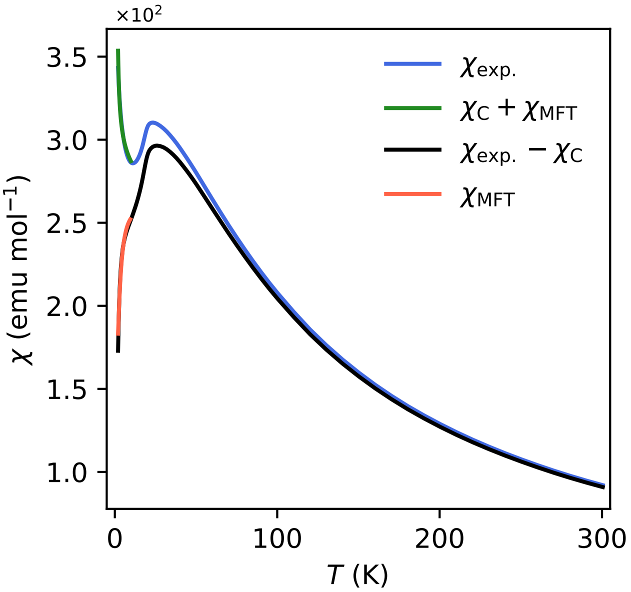

Figure S13: The zero-field cooled susceptibility data, $\chi_{\mathrm{exp.}}$, are shown here in blue. The Curie-like tail in these data were fit below $T = 10$ K by an additive Curie and mean-field theory function, $\chi_{\mathrm{C}} + \chi_{\mathrm{MFT}}$, shown in green.[3] The Curie component from this fit was then subtracted over $T = 2$ K to $T = 300$ K, $\chi_{\mathrm{exp.}} - \chi_{\mathrm{C}}$, to show the contribution of short range correlations to $\chi(T)$ more clearly (black). The mean-field theory function component of the fit, χ_{MFT}, is shown in red.

Table S2: Refined lattice parameters from powder XRD.

MW (g mol^{-1})	202.99
Crystal system	Monoclinic
Space group	$P\,1\,2_1/m\,1$
a (Å)	3.7179(2)
b (Å)	12.1304(5)
c (Å)	7.1159(5)
β(°)	94.364(5)
V (Å^3)	319.10(3)
T (K)	295
Z	2
R_{wp}	10.195
GOF	1.23785207
R_{Bragg}	4.431
Radiation	$\lambda = 1.5406$ Å

Table S3: Summary of PBE+U+MBD* geometry optimisation results using two different pseudo-potentials and a range of Hubbard U values. Ultrasoft pseudo-potentials have been used as well as NCP19 Vanderbilt (ONCVPSP) "on-the-fly" optimized norm-conserving pseudo-potentials. A cut-off energy of 1100 eV has been used with 18 k-points and a Gaussian smearing scheme with a 0.2 eV smearing width. The Broyden density-mixing scheme has been used throughout the optimisations.

	U value (eV)	Volume (Å^3)	Density (amu/Å^3)	Lattice Parameters (Å)			Cell Angles (°)			Integrated |Spin-density| ($\hbar$/2)	Cr-Cl Bond Lengths (Å)			
				a	b	c	α	β	γ		Cr atom 1		Cr atom 2	
Experimental		312.75	1.30	3.67	12.10	7.06	90.0	94.2	90.0		2.40	2.76	2.40	2.76
Ultrasoft Pseudo-potential	0	297.68	1.36	3.53	11.87	7.10	90.0	88.7	89.6	7.9	2.56	2.40	2.61	2.38
	2	312.45	1.30	3.72	12.05	7.03	90.0	97.2	89.6	8.7	2.39	2.87	2.40	2.86
	3	313.33	1.30	3.73	12.10	7.00	90.0	97.4	89.5	8.7	2.41	2.87	2.42	2.86
	4	316.06	1.28	3.77	12.10	6.97	90.0	97.5	89.5	8.8	2.42	2.88	2.42	2.88
Norm Conserving Pseudo-potential	0	255.35	1.59	3.24	11.75	6.70	90.0	91.0	89.9	7.3	2.38	2.39	2.40	2.38
	3	272.08	1.49	3.46	11.90	6.64	89.9	97.8	89.7	8.7	2.39	2.73	2.40	2.72
	4	272.03	1.49	3.47	11.90	6.64	89.9	97.8	89.7	8.7	2.40	2.73	2.40	2.73
	5	273.86	1.48	3.47	11.90	6.68	89.8	97.8	89.7	8.8	2.40	2.75	2.41	2.75
	6	275.83	1.47	3.50	12.00	6.65	89.9	98.2	89.7	8.9	2.42	2.76	2.43	2.76
	10	283.03	1.43	3.53	12.10	6.72	89.9	98.6	89.8	9.3	2.46	2.82	2.47	2.82

References

[1] A. A. Coelho, *Journal of Applied Crystallography*, 2018, **51**, 210–218.
[2] M. J. Rutter, *Computer Physics Communications*, 2018, **225**, 174–179.
[3] D. C. Johnston, *Physical Review B*, 2015, **91**, 064427.
